# Микроцефалическая остеодиспластическая первичная карликовость II типа с гипергонадотропным гипогонадизмом у 16-летнего пациента

**DOI:** 10.14341/probl13554

**Published:** 2026-07-22

**Authors:** В. В. Платонов, Е. Д. Носкова, Ю. Л. Скородок, Е. В. Плотникова, Е. Н. Суспицын, Т. В. Яковлева, Ф. З. Цораева, М. А. Полянская, Ш. А. Джамиева

**Affiliations:** Детский городской многопрофильный клинический центр высоких медицинских технологий им. К.А. Раухфуса Россия; Children’s municipal multi-specialty clinical center of high medical technology named after K.A. Rauhfus Russian Federation; Детский городской многопрофильный клинический центр высоких медицинских технологий им. К.А. Раухфуса; Санкт-Петербургский государственный педиатрический медицинский университет Россия; Children’s municipal multi-specialty clinical center of high medical technology named after K.A. Rauhfus; Saint-Petersburg State Pediatric Medical University Russian Federation; Санкт-Петербургский государственный педиатрический медицинский университет Россия; Saint-Petersburg State Pediatric Medical University Russian Federation

**Keywords:** инсулинорезистентность, сахарный диабет, MOPDII, PCNT, мутация, гипогонадизм, insulin resistance, diabetes mellitus, MOPDII, PCNT, mutation, hypogonadism

## Abstract

Микроцефалическая остеодиспластическая первичная карликовость II типа (MOPDII) представляет собой одну из форм примордиального нанизма, которая характеризуется экстремально низким ростом, микроцефалией, специфическим фенотипом, челюстно-лицевыми дисморфиями, скелетной дисплазией, нарушениями углеводного обмена, нейроваскулярными аномалиями. В данной статье описан пациент с фенотипом синдрома Секкеля, имеющий аневризмы левой внутренней сонной артерии с внутримозговыми кровоизлияниями, тромбоцитоз, артериальную гипертензию и сахарный диабет вследствие инсулинорезистентности, подтвержденной низким индексом Matsuda. На терапии метформином удалось достичь целевых показателей углеводного обмена. Клиническое секвенирование экзома выявило два редких гетерозиготных варианта в гене PCNT: c.6220C>T (p.Gln2074*) и c.4564-12T>A. Сопоставление результатов молекулярно-генетического исследования и фенотипа пациента позволило верифицировать диагноз: MOPDII. Гипергонадотропный гипогонадизм описан впервые при данном синдроме.

## Abstract (en)

Microcephalic osteodysplastic primary dwarfism type II (MOPDII) is a form of primordial nanism characterized by extreme short stature, microcephaly, specific phenotype, maxillofacial dysmorphisms, skeletal dysplasia, disorders of carbohydrate metabolism, and neurovascular abnormalities. This article describes a patient with a Seckel syndrome phenotype presenting with left internal carotid artery aneurysms with intracerebral hemorrhages, thrombocytosis, arterial hypertension, and diabetes mellitus due to insulin resistance confirmed by a low Matsuda index. Target carbohydrate metabolism values were achieved on metformin therapy. Clinical exome sequencing revealed two rare heterozygous variants in the PCNT gene: c.6220C>T (p.Gln2074*) and c.4564-12T>A. Comparison of the results of the molecular genetic study and the patient’s phenotype allowed us to verify the diagnosis of MOPDII. Hypergonadotropic hypogonadism was described for the first time in this syndrome.

Микроцефалическая остеодиспластическая первичная карликовость II типа (MOPDII) представляет собой одну из форм примордиального нанизма с аутосомно-рецессивным типом наследования. Заболевание орфанное, популяционная частота не установлена, описано около 150 случаев подтвержденного MOPDII [1].

Majewski F. et al (1982 г.) впервые описали пациентов с MOPDII, фенотипически схожих с синдромом Секкеля, но имеющих специфические изменения костей: непропорционально короткие конечности, брахимезофалангия, брахиметакарпия, V-образное расширение дистальных метафизов бедренных костей, высокий и узкий таз, эпифизиолиз проксимальных эпифизов бедренных костей и варусная деформация тазобедренного сустава [2]. Rauch A. et al (2008 г.) опубликовали данные о генетических механизмах MOPDII. Ген PCNT (21q22.3) кодирует синтез белка перицентрина, участвующего в формировании веретена деления при митозе. Мутации, приводящие к потере функции перицентрина, вызывают нарушение пролиферации клеток, что объясняет уменьшение их количества и значительное замедление роста [3]. Гены, такие как CENPJ, CEP152, CEP63, NIN, вызывающие синдром Секкеля, также кодируют центросомный белок, принимающий участие в делении клеток [4]. Поэтому эти два заболевания похожи фенотипически, но имеют разную генетическую основу. Это сходство затрудняет постановку диагноза и обуславливает необходимость проведения молекулярно-генетического исследования (МГИ) с целью идентификации мутации в гене PCNT. Несмотря на то, что MOPDII является генетически однородным заболеванием, имеются данные, что местоположение и тип мутации связаны с тяжестью клинического фенотипа [5]. В таблице 1 представлены клинические симптомы MOPDII.

**Table:** Таблица 1. Клинические симптомы микроцефалической остеодиспластической первичной карликовости II типа (Duker A.L. et al, 2021, с изменениями) Примечания: ДМПП — дефект межпредсердной перегородки; ДМЖП — дефект межжелудочковой перегородки; ООО — открытое овальное окно; ИМ — инфаркт миокарда; ППР — преждевременное половое развитие; СПЯ — синдром поликистозных яичников; СД — сахарный диабет.

Симптом	Частота встречаемости, % встречаемости
Пре- и постнатальная задержка роста	~100
Микроцефалия	~100
Скелетная дисплазия	~100
Зубные аномалии	~100
Лабораторные изменения:	
Анемия	~75
Тромбоцитоз	~75
Гиперхолестеринемия	~32
Нейроваскулярная патология:	
Аневризмы	~50
Болезнь Moyamoya	~50
Сердечно-сосудистая патология:	
Артериальная гипертензия	~43
Пороки развития сердца (ДМПП/ДМЖП/ООО)	~28
Ишемическая болезнь сердца/ИМ	~17
Нарушения репродуктивной системы:	
Крипторхизм/гипоспадия	~44%/~8
ППР/СПЯ у девочек	~29%/~1
Нарушения углеводного обмена:	
Инсулинорезистентность/СД	~38
Почечная патология:	
Хроническая болезнь почек	~32
Добавочные почечные артерии	~15
Стеноз почечной артерии/аневризма	~4

Патогенетические механизмы, лежащие в основе других фенотипических проявлений, остаются не до конца изученными. Исследуя молекулярные причины инсулинорезистентности (ИР) при MOPDII, выяснили, что транспорт глюкозы был снижен на 50% в клетках с подавленной экспрессией перицентрина, что приводит к нарушению пролиферации клеток, адипогенезу и накоплению триглицеридов в преадипоцитах. Выявить конкретные причины нарушения транспорта глюкозы в клетку in vitro невозможно; необходимы дополнительные исследования in vivo, которые помогут понять патогенез ИР при MOPDII и разработать патогенетическую терапию [6]. Другие компоненты синдрома, такие как аномалии сосудов, патология почек, тромбоцитоз, нарушения репродуктивной системы, до сих пор остаются не объясненными.

## ОПИСАНИЕ СЛУЧАЯ

Пациент 16 лет 1 месяца поступил экстренно в ДГМКЦ ВМТ им. К.А. Раухфуса переводом из другого стационара в связи с гипергликемией 18–20 ммоль/л после хирургического лечения множественных внутримозговых кровоизлияний (аневризма левой внутренней сонной артерии, ЛВСА). Отмечался эпизод повышения АД до 160/80 мм рт.ст. Пациент от первой беременности (угроза прерывания на всем протяжении), родов на 33‑й неделе путем кесарева сечения с массой тела 1040 г (SDS -2,19), длиной 33 см (SDS -4,0) — малый для гестационного возраста. С первых дней жизни получал трансфузии эритроцитарной массы в связи с тяжелой анемией, в 2 месяца оперирован по поводу некротизирующего энтероколита. В 8 месяцев диагностирован врожденный порок сердца (ВПС): дефект межпредсердной перегородки (ДМПП, гемодинамически значимый, НК I ст.), стеноз легочной артерии (ЛА). В 4 года при катетеризации бедренной артерии (подготовка к хирургическому лечению ВПС) развился острый тромбоз бедренной артерии справа. В 9 лет закрытый перелом средней трети правой бедренной кости со смещением. Наблюдался у эндокринолога с диагнозом «синдром Секкеля» (МГИ не проводили), а также у невролога (гипоксическое поражение ЦНС, синдром двигательных нарушений, темповая задержка психоречевого развития, микроцефалия), ортопеда (плосковальгусная постановка стоп, кифоз, сгибательные контрактуры локтевых суставов, укорочение правой конечности), офтальмолога (миопия слабой степени, двусторонний хориоретинит). Наследственный анамнез неизвестен.

В 16 лет 1 месяц (рис. 1) рост — 97 см (SDS -9,81), вес — 15,05 кг, ИМТ — 16,0 кг/м² (SDS -2,37). Подкожно-жировая клетчатка истончена, распределена равномерно. Кифоз, сколиоз, бочкообразная грудная клетка. Сгибательные контрактуры локтевых суставов. Укорочение правой конечности. Плоско-вальгусная деформация стоп, синдактилия IV–V пальцев правой стопы, аплазия III пальца левой стопы. Выраженные множественные черепно-лицевые дисморфии: уплощенный затылок, микроцефалия, клювовидный нос, микрогнатия, дисплазия и неправильное расположение зубов, низко расположенные ушные раковины, короткая широкая шея. Признаки ИР: черный гиперкератоз шеи, аксиллярных областей, «вельветовая» кожа. Диспигментация кожи: чередуются участки гипо- и гиперпигментации (пятна цвета кофе с молоком) неправильной формы, различной величины. АД 127/65 мм рт.ст. Мышечная гипотония. Половое развитие по Tanner PII, правая тестикула в паховом канале, низводится в мошонку ~2–3 мл, левая тестикула в мошонке и паховом канале не пальпируется, половой член 5×1,3 см, синехии крайней плоти.

**Figure:**
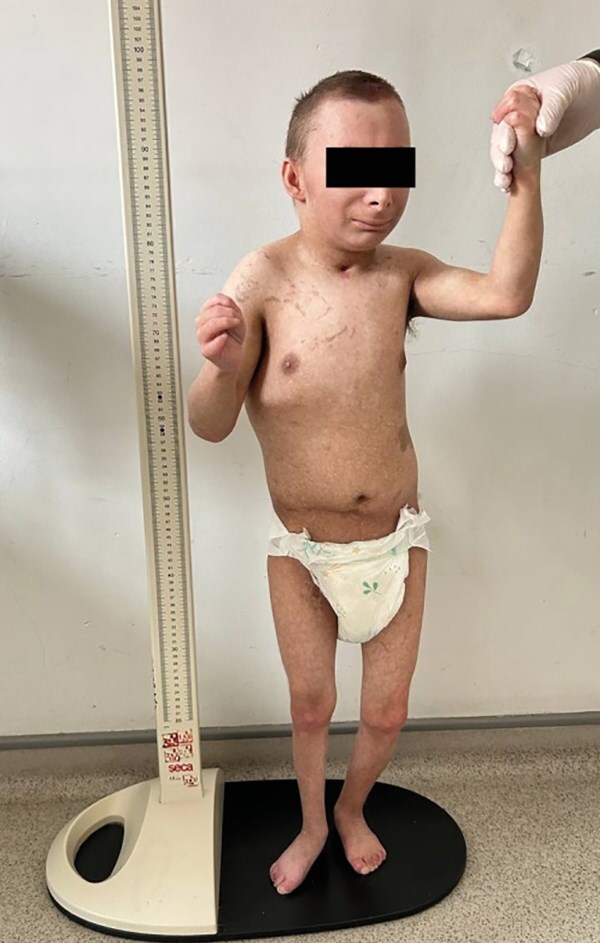
Рисунок 1. Пациент 16 лет 1 месяц с микроцефалической остеодиспластической первичной карликовостью II типа.

Выявлены анемия (гемоглобин 69–116 г/л; норма 130–160), эритропения (2,12–3,8×10¹²/л; норма 4,00–5,10), тромбоцитоз (711–371×10⁹/л; норма 150–400), дислипидемия (триглицериды — 2,34 ммоль/л; норма 0,42–1,67, ЛПВП — 0,81 ммоль/л; норма ≥1), повышенный HbA1c (6,7%; норма ≤6,0%, диагностический для СД ≥6,5%).

Функция щитовидной железы и надпочечников не нарушена (ТТГ — 1,16 мМЕ/л; норма 0,4–4,0, свТ4 — 18,73 пмоль/л; норма 10–23, АКТГ — 3,4 пмоль/л; норма 0–9,2, кортизол — 365,7 нмоль/л; норма 83–580). Повышены уровни гонадотропных гормонов (ФСГ — 20,0 МЕ/л; норма 1,53–15,4, ЛГ — 19,25 МЕ/л; норма 0,56–7,8) при допубертатном — общего тестостерона (3,43 нмоль/л; норма 3,64–18,91). УЗИ мошонки: правое яичко в нижней трети пахового канала, эхогенность обычная, структура неоднородная, с точечными гиперэхогенными включениями, контуры четкие, ровные, объем — 0,8 см³; придаток достоверно не визуализируется. Левое яичко в малом тазу при входе в паховый канал, эхогенность обычная, структура неоднородная, с точечными гиперэхогенными включениями, контуры четкие, ровные, объем: 1,3 см³; придаток достоверно не визуализируется. Заключение: объем яичек значительно уменьшен. УЗ-картина тестикулярного микролитиаза с обеих сторон. Гипергонадотропинемия у пациента с крипторхизмом и уменьшенным объемом тестикул позволили заподозрить гипергонадотропный гипогонадизм. Концентрация ИФР-1 211,6 нг/мл, (SDS -1,78 для возраста и пола).

На основании хронической гипергликемии и диабетического HbA1c диагностирован СД, потребовавший инсулинотерапии (ИТ) внутривенно микроструйно в дозе 0,1–0,2 ЕД/кг/час с последующим переводом на подкожное введение инсулина: Гларгин 10 ЕД перед сном и Аспарт 5–7 ЕД перед основными приемами пищи с дальнейшей коррекцией.

Динамика гликемии на фоне внутривенного и подкожного введения инсулина представлена на рис. 2 и 3.

**Figure:**
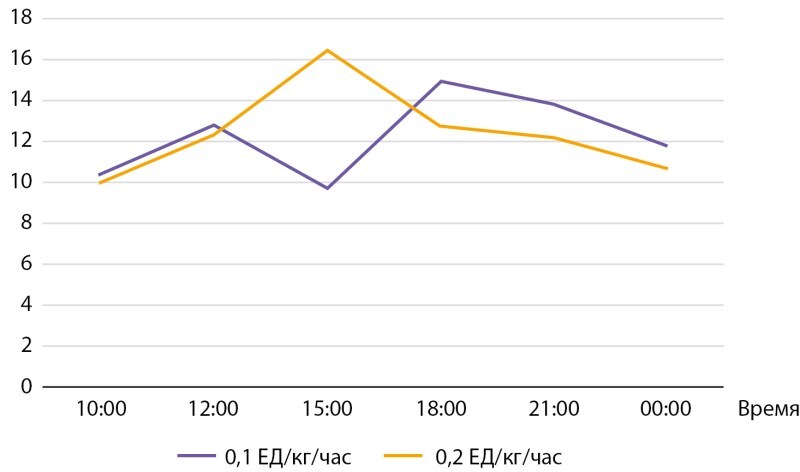
Рисунок 2. Динамика гликемии на фоне внутривенного введения инсулина.

**Figure:**
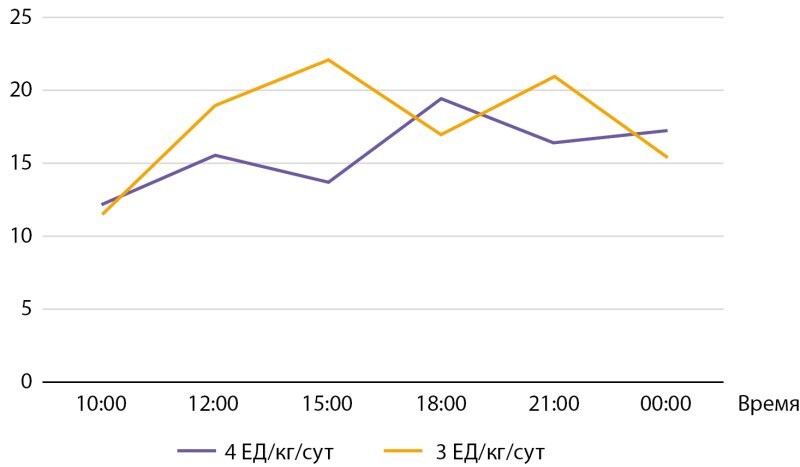
Рисунок 3. Динамика гликемии на фоне подкожного введения инсулина.

Как видно из рис. 2, 3, ИТ была неэффективна (отмечалась стойкая гипергликемия 10–24 ммоль/л) независимо от пути введения препарата и несмотря на суточную дозу до 4 ЕД/кг. Учитывая неэффективность ИТ у пациента с клиническими признаками ИР, инсулин был отменен и на «чистом» фоне определен уровень инсулина натощак (70 мкЕд/мл; норма 2,6–24,9) и в ходе 3-часового перорального глюкозотолерантного теста, рассчитан индекс Mastuda, который составил 0,56 (норма ≥2,6). Назначен метформин в начальной дозе 500 мг/сут с последующей титрацией до 2000 мг/сут.

Динамика гликемии на фоне терапии метформином представлена на рис. 4.

**Figure:**
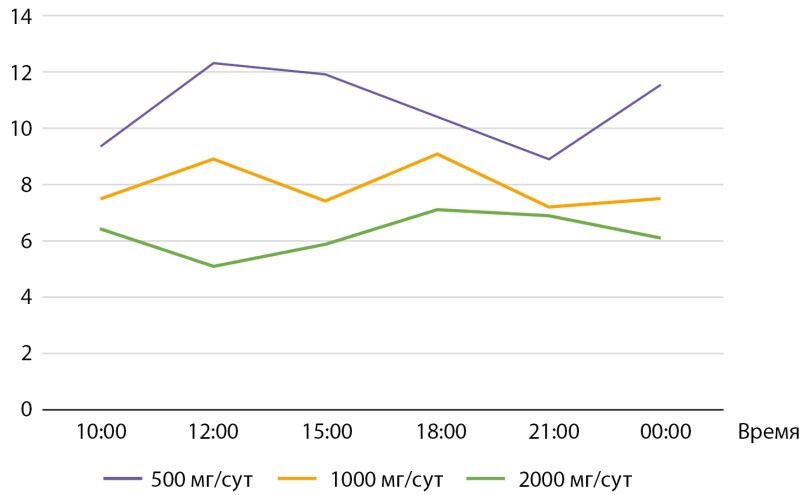
Рисунок 4. Динамика гликемии на фоне терапии метформином.

Как видно из рис. 4, на дозе метформина 500 мг/сут, отмечалась умеренная гипергликемия 9–12,2 ммоль/л. При увеличении дозы до 1000 мг/сут уровни глюкозы снизились до 7,5–9 ммоль/л. На дозе 2000 мг/сут удалось достичь целевой гликемии 5–7 ммоль/л.

На Эхо-КГ подтвержден ДМПП в средней трети 5,3 мм. Данных за стеноз ЛА не получено. При КТ головного мозга с контрастом определяются мешотчатые аневризмы диаметром 0,3 см и 0,5 см ЛВСА.

В возрасте 16 лет 5 месяцев на дозе метформина 1000 мг/сут уровень глюкозы крови натощак 4,97 ммоль/л (целевой 3,6–5,6), HbA1c 5,4% (целевой ≤6,5%), инсулин 8,65 мкЕд/мл (норма 2,6–24,9). Сохраняется гипергонадотропинемия (ФСГ 32,8 МЕ/л; норма 1,53–15,4 ЛГ 16,46 МЕ/л; норма 0,56–7,8) при сниженном уровне общего тестостерона (3,56 нмоль/л; норма 3,64–18,91), что подтверждает наличие гипергонадотропного гипогонадизма.

Учитывая характерный фенотип, включая крайне тяжелую первичную низкорослость с микроцефалией и дисплазией скелета, признаки ИР, аневризмы ЛВСА, тромбоцитоз, артериальную гипертензию (АГ), заподозрена синдромальная форма примордиального нанизма, отличная от синдрома Секкеля (MOPDII?). Клиническое секвенирование экзома (TruSight One Sequencing Panel, Illumina) позволило выявить два редких гетерозиготных варианта гена PCNT (транскрипт NM_006031.6) в компаунде: c.6220C>T (p.Gln2074*) и c.4564-12T>A. Первый из них расценивается согласно критериям ACMG как вероятно патогенный, второй, ранее не описанный — как вариант с неизвестным клиническим значением.

## ОБСУЖДЕНИЕ

У пациента с фенотипом синдрома Секкеля отмечались нейроваскулярная патология (аневризмы ЛВСА), СД на фоне выраженной ИР, тромбоцитоз, АГ. Данные клинические проявления ранее описаны другими авторами у пациентов с генетически подтвержденным синдромом MOPDII. Так, нейроваскулярная патология отличает MOPDII от других форм синдромальной низкорослости. Duker A.L. et al (2021) на основании анализа медицинской документации 47 пациентов выявили у 22 (47%) в возрасте 6 месяцев–24 лет прогрессирующее заболевание внутренних сонных артерий с сужением просвета вплоть до полной окклюзии (болезнь Moyamoya), у 25 (53%) — аневризмы головного мозга [7]. В описанном случае у пациента выявлены мешотчатые аневризмы диаметром 0,3 см и 0,5 см ЛВСА, которые привели к множественным внутримозговым кровоизлияниям, потребовавшим хирургического лечения. Данных за болезнь Moyamoya не получено.

АД у представленного пациента (127/65 мм рт.ст.) превышало 95 перцентиль для роста (106/66) [8], что позволило диагностировать АГ, характерную для синдрома MOPDII, и назначить антигипертензивную терапию. Исследователи предполагают наличие связи между АГ при MOPDII и нейроваскулярной и/или реноваскулярной патологией, взаимно отягощающих друг друга [7].

У пациента гипергликемию впервые выявили в 16 лет 1 месяц на фоне хирургического лечения множественных внутримозговых кровоизлияний. Отсутствие эффекта от инсулина и клинические эквиваленты ИР вынудили прервать ИТ и, после лабораторного подтверждения ИР (индекса Mastuda 0,56), назначить метформин. Литературные данные указывают на связь ИР с MOPDII, хотя генез ИР остается неясен. Так, Huang-Doran I et al., (2011) среди 21 пациента с генетически подтвержденным MOPDII у 18 (85%) выявили клинические и/или лабораторные признаки ИР, у 10 (47%) в возрасте 5–28 лет — СД. При этом у 2 пациентов клинические и/или лабораторные маркеры ИР впервые обнаружены на фоне терапии препаратами рекомбинантного гормона роста, в связи с чем их применение при MOPDII не рекомендуется [6].

У представленного пациента выявлен тромбоцитоз, который, вероятно, присутствовал давно, учитывая эпизод острого тромбоза бедренной артерии справа при ее катетеризации в возрасте 4 лет. Это согласуется с литературными данными о связи тромбоцитоза при синдроме MOPDII и подобными осложнениями при инвазивных манипуляциях [7]. Кроме того, для MOPDII характерна гиперхолестеринемия [9], которой у нашего пациента не было, но наличие гипертриглицеридемии и снижения ЛПВП свидетельствует о дислипидемии как дополнительном факторе риска декомпенсации сосудистых заболеваний.

В описанном нами случае MOPDII у пациента с крипторхизмом и допубертатными размерами тестикул гипергонадотропинемия указывала на наличие гипергонадотропного гипогонадизма. По литературным данным, крипторхизм встречается почти у половины пациентов, однако сведений об уровнях гонадотропных и половых гормонов при данном синдроме не найдено.

Учитывая клинические симптомы, не характерные для синдрома Секкеля, у пациента была заподозрена MOPDII. Для уточнения диагноза проведено клиническое секвенирование экзома, в результате которого выявлено два редких варианта PCNT в гетерозиготном состоянии (компаунд-гетерозигота): вероятно патогенный и имеющий неизвестное клиническое значение (VUS). Сопоставление результатов МГИ с фенотипом пациента позволяет считать диагноз MOPDII подтвержденным.

## Заключение

У пациента с сочетанием фенотипа синдрома Секкеля и нейроваскулярных аномалий (аневризмы сосудов головного мозга), сахарного диабета вследствие инсулинорезистентности, тромбоцитоза, артериальной гипертензии молекулярно-генетическое исследование подтвердило диагноз микроцефалической остеодиспластической первичной карликовости II типа идентификацией биаллельной мутации в гене PCNT. Терапия метформином позволила достичь целевых показателей углеводного обмена. Особенностью представленного случая стало наличие гипергонадотропного гипогонадизма, ранее не описанного при данном синдроме.

## Дополнительная информация

Источники финансирования. Исследование выполнено за счет средств гранта РНФ № 24-45-00067.

Конфликт интересов. Авторы декларируют отсутствие явных и потенциальных конфликтов интересов, связанных с содержанием настоящей статьи.

Участие авторов. Все авторы одобрили финальную версию статьи перед публикацией, выразили согласие нести ответственность за все аспекты работы, подразумевающую надлежащее изучение и решение вопросов, связанных с точностью или добросовестностью любой части работы.

Согласие пациента. Пациент добровольно подписал информированное согласие на публикацию персональной медицинской информации.
